# Energy Intake, Macronutrient Profile and Food Sources of Spanish Children Aged One to <10 Years—Results from the EsNuPI Study †

**DOI:** 10.3390/nu12040893

**Published:** 2020-03-25

**Authors:** Casandra Madrigal, María José Soto-Méndez, Ángela Hernández-Ruiz, Teresa Valero, José Manuel Ávila, Emma Ruiz, Federico Lara Villoslada, Rosaura Leis, Emilio Martínez de Victoria, José Manuel Moreno, Rosa M. Ortega, María Dolores Ruiz-López, Gregorio Varela-Moreiras, Ángel Gil

**Affiliations:** 1 Department of Nutrition and Bromatology, Faculty of Pharmacy, University of Granada, Campus de Cartuja, s.n, 18071 Granada, Spain; casandram@correo.ugr.es; 2 Iberoamerican Nutrition Foundation (FINUT), Av. Del Conocimiento 12, 3 ª pta, Armilla, 18016 Granada, Spain; msoto@finut.org (M.J.S.-M.); ahernandez@finut.org (Á.H.-R.); agil@ugr.es (Á.G.); 3 Spanish Nutrition Foundation (FEN), c/General Álvarez de Castro 20, 1ªpta, 28010 Madrid, Spain; tvalero@fen.org.es (T.V.); jmavila@fen.org.es (J.M.Á.); e.ruiz@externos.isciii.es (E.R.); gvarela@ceu.es (G.V.-M.); 4 CIBERESP, Consortium for Biomedical Research in Epidemiology and Public Health, Carlos III Health Institute, 28029 Madrid, Spain; 5 National Center for Epidemiology, Carlos III Health Institute, 28029 Madrid, Spain; 6 Instituto de Nutrición Puleva, Camino de Purchil 66, 18004 Granada, Spain; federico.lara@lactalis.es; 7 Unit of Pediatric Gastroenterology, Hepatology and Nutrition, Department of Pediatrics, University Clinical Hospital of Santiago, IDIS, ISCIII, University of Santiago de Compostela, 15700 Santiago de Compostela, Spain; mariarosaura.leis@usc.es; 8 CIBEROBN (Physiopathology of Obesity and Nutrition CB12/03/30038), Instituto de Salud Carlos III (ISCIII), 28029 Madrid, Spain; 9 Department of Physiology, Faculty of Pharmacy, University of Granada, Campus de Cartuja, s.n, 18071 Granada, Spain; emiliom@ugr.es; 10 Institute of Nutrition and Food Technology “José Mataix,” Biomedical Research Center, University of Granada, Parque Tecnológico de la Salud, Avenida del Conocimiento s/n, Armilla, 18100 Granada, Spain; 11 Pediatric Department, University of Navarra Clinic, Calle Marquesado de Sta. Marta, 1, 28027 Madrid, Spain; jmorenov@unav.es; 12 Department of Nutrition and Food Science, Faculty of Pharmacy, Complutense University of Madrid, Plaza Ramón y Cajal s/n, 28040 Madrid, Spain; rortega@ucm.es; 13 Department of Pharmaceutical and Health Sciences, Faculty of Pharmacy, CEU San Pablo University, Urb. Montepríncipe, crta. Boadilla km. 5.3, Boadilla del Monte, 28668 Madrid, Spain; 14 Department of Biochemistry and Molecular Biology II University of Granada, University of Granada, Campus de Cartuja, s.n, 18071 Granada, Spain

**Keywords:** energy intake, food sources, EsNuPI study, pediatrics, Spanish children, misreporting, feeding behavior, dietary habits, nutrition assessment, pediatric nutrition

## Abstract

The present study aimed to assess energy intake, nutrient profile and food sources in Spanish children participating in the EsNuPI (“Estudio Nutricional en Población Infantil Española”) study. Plausibility of energy intake and adequacy of nutrient intakes to international recommendations were analyzed in a final sample of 1448 subjects (728 boys and 720 girls) and one group representative of the 1 to <10 years old urban Spanish children (reference sample (*n* = 707)) who consumed milk and one of the same age who consumed adapted milk over the last year (adapted milk consumers sample (*n* = 741)) were compared. Both groups completed data of a face-to-face and a telephone 24-h dietary recalls. Both the reference and the adapted milk consumers samples reported an adequate daily energy intake (1503 kcal/day and 1404 kcal/day); and a high contribution to total energy from protein (16.5% and 15.6%) and fat (36.5% and 35.9%). Also, a high percentage of children from both samples were below the lower limit of the recommendations for carbohydrates (47.8% and 39.3%). As the percentage of plausible energy reporters was high for both groups (84.7% and 83.5%, respectively), data for the whole sample were analyzed. Milk and dairy, cereals, meat and derived products, fats and oils, bakery and pastry, fruits and vegetables contributed to about 80% of the total energy intake in both groups. However, the reference sample reported significantly more contribution to energy from cereals, meat and meat products, bakery and pastry and ready to cook/eat foods; meanwhile, the adapted milk consumers sample reported significantly more energy from milk and dairy products, fruits and eggs. Those results suggest that adapted milk consumers have better adherence to the food-based dietary guidelines. Further analyses are warranted to characterize food patterns and the quality of the diet in the EsNuPI study population.

## 1. Introduction

Infancy and childhood are both critical periods of rapid physical growth and cognitive development [1]. At this stage of life, children have high nutrients needs; consequently, the right amount of dietary energy intake and the consumption of nutrient-rich foods are fundamental [2].

It is very important to provide a good variety and diversity of foods for each stage of age to ensure child development [3]. The Global Burden of Disease (2017) concluded that poor dietary habits are associated with a range of chronic diseases and can potentially be a major contributor to chronic noncommunicable diseases (NCD) and a cause of mortality in all countries worldwide (11 million deaths in 2017) [4]. For example, regular consumption of dairy products, especially cow’s milk (CM) (which is one of the most often consumed dairy products by young children worldwide) is an important determinant of childhood development due to their rich nutrient profile which includes macro (protein, fat) and micronutrients (calcium, vitamin D) that help to maintain good health [5,6].

Despite the nutritional and dietary importance of milk and dairy products, consumption is decreasing and moving away from the advised level in many countries, including Spain [5]. Furthermore, for some time, the potential benefits of milk and dairy products for health have come under question [7]. However, Santaliestra-Pasías et al. (2019) suggest that European children with a healthier lifestyle consumed more milk and yogurt and Ortega et al. concluded that the Spanish children who drank more milk also had better dietary patterns [8,9].

Nonetheless, according to the European Food Safety Authority (EFSA) recommendation, young child formula (YCF) is an alternative to CM or breast milk for children 1-3 years of age. It has been suggested as a resource to help to achieve adequate intakes of critical nutrients in young children. They are fortified with several nutrients, including iron, vitamin D and essential fatty acids (EFA) and they contain less protein, saturated fat and sodium than CM [10]. Also, according to the European Society for Pediatric Gastroenterology, Hepatology and Nutrition (ESPGHAN) the YCF can be used as part of a strategy to increase the intake of iron, vitamin D and n-3 PUFA and decrease the intake of protein compared with unfortified CM [11].

In Spain, the adapted and fortified milk formulas have an important market share. According to Kantar World Panel (T4 2019) adapted milk, targeted children over 12 months, have a total home penetration of 15% with 3.5% for those targeted children over 3 years. The total annual volume of infant adapted milk accounts for 57 million liters [12]. Nevertheless, currently, there is some controversy about the advantages or disadvantages of drinking YCF or CM between 1-3 years old and whether the use of fortified milk formulas in later ages represents a nutritional advantage versus the exclusive use of cow’s milk [10,11,12,13,14].

Given the above, it is essential to understand dietary nutrient intakes and nutrient food sources in children due to the fact that early life nutrition determines the type of future food preferences, eating habits and influences the future health outcomes [1,15]. Specifically, inadequate intake of energy and nutrients (such as high protein or fat intake) could have an adverse effect on children’s and adolescents’ health and predispose them to the risk of development of non-communicable diseases including overweight and obesity, cardiovascular diseases and type 2 diabetes mellitus [16,17].

During the last decades, the children’s lifestyles have changed, including dietary habits, a phenomenon called “nutritional transition”; this transition potentially changed the usual energy and macronutrient intakes in the children over the last years, having hypothetically negative consequences in the children’s health [18,19,20]. Furthermore, in Spain, the most recent studies suggest that food patterns, energy and nutrient intakes have changed noticeably in the last forty years, differing somewhat at present from the traditional and healthy Mediterranean Diet [21,22,23,24]. This is one of the reasons that could explain why Spain is listed within the countries with more childhood obesity in Europe—40% of Spanish children already have overweight or obesity [25].

Subsequently, the need to study and monitor the children’s health, diet quality, nutrients and energy intake has been increasing in Spain and many other countries. Knowledge about food and energy intake in childhood is crucial and critical as it provides an opportunity for interventions to encourage healthy habits to mitigate the occurrence of diet-related chronic diseases [18,26,27].

There are different dietary recall methods used in nutrition surveys, which are based on the assumption that reported dietary intake shows the habitual intake. The procedure of obtaining the habitual intake becomes more complex in young children, for whom dietary recall methods are conducted on proxy-reporters [28,29]. Therefore, the identification of the characteristics associated with misreporting (under- and over-reporting) of dietary intake is fundamental to obtaining results that represent the usual intake and that is physiologically plausible [30]. In 2013 the EFSA published the “Guidance on menu methodology in the European Union,” a document developed to facilitate the comparison of intake data among European countries and to determine the quality of the dietary information collected in nutritional studies [31].

Several national and international studies investigate children’s diet and food sources of energy intake. In Spain, the “Alimentando la Salud del Mañana” (ALSALMA) study, carried out in 2013 (*n* = 1320), assessed the nutritional patterns of children under three years of age (0-36 months) [24]. However, as physical activity data were not collected, it was not possible to estimate the misreporting of energy intake. The National Dietary Survey on the Child and Adolescent Population project in Spain (ENALIA) study (*n* = 1862) carried out in 2013–2014 was designed to collect dietary intake and information of dietary habits in Spanish children and adolescents. Even though, this study only collected the physical activity of children ≥10 years old and assumed the physical activity levels estimated by EFSA to calculate misreporting [22,31]. Neither of the two above mentioned studies analyzed the contribution of food sources to intakes of total energy. The Anthropometry, Intake and Energy Balance Study (ANIBES) analyzed the energy intake and the food and beverage sources that contributed to energy intake but this was carried out only in children 9 to 12 years old (*n* = 213) [23].

In addition, the Identification and prevention of Dietary- and lifestyle-induced health EFfects In Children and infantS (IDEFICS) study (*n* = 9560) evaluated the energy intake and food sources in European children aged 2 to 9 years old; however, this study did not include a representative sample of the Spanish population [18].

Finally, The National Health and Nutrition Examination Survey (NHANES) 2011-2014 study (*n* = 5876) in children aged two-18 years old assessed the misreporting of the energy intake and the dietary sources of total energy and nutrients in a nationally representative sample of United States. Nonetheless, to determine the misreporting, this study assumed the physical activity because of the lack of an objective measure for this variable [32].

Based on the identified gap of complete information related to food intake, physical activity and lifestyle behaviors of Spanish children, the aim of the present study was to assess energy intake, nutrients profile, food sources and plausibility of energy intake, based on physical activity level, reported by Spanish children and to compare data from the two study subsamples, one representative of the one to <10 years of urban Spanish children (SRS) consuming milk and one of adapted milk consumers (AMS) of the same age living in urban areas and regularly consuming adapted and fortified milk formulas, participants in the EsNuPI (from the Spanish “Estudio Nutricional en Población Infantil Española”) study.

## 2. Materials and Methods

### 2.1. Study Design and Sample

The data used in this work were obtained as part of the EsNuPI study, which is a prospective, cross-sectional, observational study, conducted from October 2018 to January 2019. The complete design, protocol and methodology of the EsNuPI study have been described in detail elsewhere [33].

Briefly, EsNuPI study evaluated the dietary and nutrient intake and dietary patterns, as well as physical activity and sedentary behaviors of Spanish children (excluding the autonomous cities of Melilla and Ceuta), living in urban areas with >50,000 inhabitants, distributed in nine regions according to Nielsen Spanish areas. Two subsamples were selected, one representative of the urban non-vegan Spanish population (SRS) from one to <10 years old consuming milk in the last 12 months and one called “adapted milk consumers” (AMS) of non-vegan Spanish population from one to <10 years old also living in urban areas and consuming adapted and fortified milk formulas over the last 12 months.

For this study, the following were considered as “milk formulas”—infant formula, follow-on milk formula, toddler’s milk formula (also termed “young children milk formula” and in Spain “growing up” milk formula) and fortified milk formula (e.g., docosahexaenoic acid (DHA), calcium, vitamin D, iron).

A total of 1514 children (*n*= 742 SRS; *n*= 772 AMS) agreed to participate in the study and completed the face-to-face interview providing sociodemographic information, answering a quantitative food frequency questionnaire (FFQ), a physical activity and sedentary behaviors questionnaire (PABQ) and the first 24-h dietary recall (24-h DR).

The EsNuPI study was conducted in accordance with the declaration of Helsinki and was approved by the University of Granada ethical committee (No. 659/CEIH/2018) and registered in ClinicalTrials.gov (Unique Protocol ID: FF01/2019).

### 2.2. Dietary Survey and Data Collection

One face-to-face and one telephone 24-h DR were completed on non-consecutive days, including weekdays and weekend days, using the parents or caregivers as a proxy to determine children’s dietary intake.

The participant made a detailed description of the dietary intake (ingredients, method of preparation and brands); this information allowed the correct coding and weight assignment for each food item. The information obtained was structured as mealtimes (breakfast, mid-morning, lunch, mid-afternoon, dinner and other moments), which helped us to calculate the distribution of energy and nutrients in the different moments of the day.

As support material, the interviewers used the “Tables of common home measures and habitual portion sizes for Spain population” [34,35] and the “Photo guide of common portions sizes of Spanish foods” [36] built using the “pilot study for the assessment of nutrient intake and food consumption among kids in Europe” (PANCAKE) standards [37]. The photo guide includes 12 food groups, 204 foods commonly consumed in the Spanish population and 944 photographs.

Furthermore, a software called “VD-FEN 2.1” a Dietary Evaluation Program from the Spanish Nutrition Foundation (FEN) [35] was used to calculate the food, beverage and energy and nutrient reported intakes. This program is based mainly on Spanish food composition tables, with several expansions and updates [34].

Reported intake data were compared with EFSA [38] and Institute of Medicine [39] recommendations for analyzing the energy, protein, carbohydrates, total fat, saturated fatty acids, mono- and poly-unsaturated fatty acids.

Finally, the different food items were categorized into the following 18 food categories: “milk and dairy products,” “cereals,” “meat and meat products,” “oils and fats,” “bakery and pastry,” “fruits,” “vegetables,” “sugars and sweets,” “ready to cook/eat,” “other dairy products,” “beverages,” “legumes,” “eggs,” “fish and shellfish,” “appetizers,” “cereal-based baby foods and supplements,” “nuts” and “sauces and condiments.”

### 2.3. Evaluation of Plausible, Under and Over Reporters (Misreporting)

Intentional as well as unintentional misreporting, which comprises under- and over-reporting, are well-known problems in dietary assessment and maybe even more pronounced in data relying on proxy reports [18].

For assessing the misreporting of the energy intake of this study, the EFSA misreporting protocol was used [31]. This method is based on the Goldberg and Black work that defined cut-off values to classify 24-HDRs in plausible energy reports, under-reports and over-reports, respectively [40,41].

To check for misreporting, the ratio of reported energy intake (EI) and predicted Basal Metabolic Rate (BMR) was used. BMR was estimated for each subject according to the Schöfield equations based on age, sex, height and weight [42]. Height and weight data were declared by parents or caregivers based on the child’s health card.

Table 1 shows the physical activity level (PAL) values that were assigned at an individual and a group level, according to the EFSA recommendations [31] and determined by adding up the total metabolic equivalents (MET_y_), of all activities collected through a physical and sedentary behavior questionnaire adapted from Camargo, et al. 2015 [43].

Subjects were identified as plausible, under- or over-reporters of energy intake based on their ratio of reported energy intake to estimated energy requirement.

The 95% lower confidence limits (± 2 standard deviation (SD) cut-offs) were calculated using the Black formula [41]. According to these criteria, children with reported EI below the cut-offs were considered as under-reporters; recalls with EIs between the cut-offs were considered plausible reporters and those with EIs above the cut-offs were considered high energy reporters or “over-reporters.”

### 2.4. Statistical Analysis

Once all dietary intake information was collected, the reported 746 food items were grouped into 18 food groups and transformed into energy and nutrient data for in-depth analysis. This information was processed using different statistical methods. The Kolmogorov-Smirnoff normality test and histogram graphs were used to determine the normality of the distribution of the variables to decide between parametric or nonparametric analyses for comparisons.

To describe energy intake by type of sample (SRS and AMS), by sex and age groups, mean, standard deviation (SD), median and interquartile range (IQR) were used for continuous variables and frequencies and percentages for categorical variables.

Every comparison by sex and age group between samples (SRS and AMS) was performed by a Student’s t-test or Mann-Whitney U-test. Analysis of variance (ANOVA) tests with Bonferroni correction for multiple comparisons or Kruskal-Wallis analysis were used to calculate differences among each age group within samples. A student´s t-test or Chi-squared test was used to evaluate adequacy differences between samples (SRS and AMS) by sex and age groups. Kruskal-Wallis or *Z*-test with Bonferroni correction were used for multiple comparisons among age groups within the samples.

All P values were two-tailed and statistical significance was considered at the 5% level (*p* < 0.05). All data analyses were performed using IBM SPSS 20.0 (IBM Corp., Armonk, NY, USA).

## 3. Results

### 3.1. Description of the Sample

A total of 1448 (95.6%) children of the EsNuPI study sample whose parents or caregivers agreed to participate and completed the 2nd 24-h DR (49.7% girls and 50.2% boys) were analyzed. The total SRS represented 48.8% and the AMS 51.1%. The sample was stratified by 3 age groups: Gp 1) 1<3 years old (31.5%), Gp 2) 3<6 years old (34.9%) and Gp 3) 6–<10 years old (33.6%). The characteristics of both study samples are given in Table 2.

### 3.2. Total Energy Intake

Mean and distribution of daily energy intakes (median and interquartile range) for the SRS and AMS by age group and sex are shown in Table 3. SRS had higher energy intake than AMS (1484 kcal and 1375 kcal, respectively) (*p* < 0.001). Similar results were observed when comparing children of the same sex between the two samples (boys *p* < 0.001 and girls *p* = 0.018). According to age groups and between both samples, no statistically significant differences were found.

Within the SRS, boys had higher energy intake than girls (1515 kcals vs. 1461 kcal) (*p* = 0.043). In the AMS, no statistically significant differences were found when comparing by sex.

When comparing among age groups for both SRS and AMS, all three age groups exhibited significantly different energy intakes (*p* < 0.05).

### 3.3. Macronutrient Profile and Distribution

In terms of the percentage of contribution of macronutrients to total dietary energy intake for the SRS and the AMS, results are shown in Table 4.

In the SRS, the carbohydrates contributed the highest proportion (45.4%) to dietary energy, followed by fat (36.5%) and proteins (16.5%). In the AMS, the carbohydrates also contributed the highest proportion (46.7%) to dietary energy intake, continued by fat (35.9%) and proteins (15.6%).

The energy contribution from proteins was lower in the AMS compared with the SRS (15.6% vs. 16.5%) (*p* < 0.001). Meanwhile, the AMS had a higher percentage of energy contribution from carbohydrates (46.7% vs. 45.5%) (*p* < 0.001). For fat, no statistically significant differences were found between AMS and MRS (*p* = 0.077). When splitting samples by sex, differences between boys and girls of the two samples were significant (*p* < 0.05).

When comparing by age groups, the AMS children of the Gp 1 and Gp 2 had a lower percentage of protein derived energy intake than the Gp 1 and Gp 2 of the SRS (*p* = 0.06 and *p* < 0.001, respectively). Besides, the children of the Gp 1 in the SRS had a lower percentage of contribution from carbohydrates to the total energy intake than the adapted milk consumers (*p* = 0.001). Finally, the percentage of contribution from fat to the energy intake in the Gp 1 of the adapted milk consumers was lower than the Gp 1 of the SRS (*p* = 0.013).

### 3.4. Adequacy to the European Food Safety Authority and the Institute of Medicine Nutrient Recommendations.

The percentage of adequacy for energy and macronutrient profile intake by age group and sex according to the EFSA [38] and Institute of Medicine (IOM) [39] recommendations are shown in Table 5.

The adequacy for energy intake, according to the EFSA recommendations, was 113% for the SRS and 120% for the AMS (*p* < 0.001). There were significant differences for girls between samples (*p* < 0.001). According to the IOM recommendations, the percentage of children that meet the energy intake was higher in adapted milk consumers than in the reference sample (93.4% vs. 84.0%) (*p* < 0.001). There were significant differences for both girls and boys between SRS and AMS (*p* < 0.001).

With regards to protein intake, the adequacy to the EFSA recommendations (g/kg/d) was 353% for the SRS and 362% for the AMS. According to the IOM recommendations, the adequacy for protein was 351% in the SRS and 342% for the AMS. The mean adequacies for protein were not significantly different between SRS and AMS groups when analyzed using both, the EFSA and the IOM recommendations. When compared to the IOM recommendations, results showed a significant difference for boys between groups (*p* = 0.005). For carbohydrates, the AMS had a higher percentage of children meeting the EFSA recommendations than the SRS (58.6% vs. 51.1%) (*p* = 0.002). There were significant differences for boys between both groups, according to EFSA recommendations (*p* = 0.024). A higher proportion of the AMS met the IOM recommendations compared with the SRS (60.6% vs. 51.9%) (*p* = 0.004). There were significant differences for girls between both groups (*p* = 0.038).

The SRS had a higher percentage of children meeting the EFSA recommendations for fat than the AMS (37.1% vs. 34.0%) (*p* < 0.001); however, a higher proportion of the AMS met the IOM recommendations for fat (49.1% vs. 45.4%) (*p* < 0.001). There were significant differences for both sexes, between both groups when compared to both recommendations (*p* < 0.05).

Finally, there were differences by age groups between both samples. More children from the AMS Gp 1 met the EFSA recommendations for fat than the Gp 1 of the SRS (58.6% vs. 51.1%) (*p* = 0.024). However, the adequacy to the EFSA recommendations for proteins in Gp 1 children from the SRS was 400% and in Gp 1 children from the AMS, it was 371% (*p* = 0.025).

A higher percentage of children in the Gp 1 from the AMS met the recommendation of the IOM for carbohydrates and fat than the children in the Gp 1 from the SRS (68.7% vs. 58.6% and 29.9% vs. 27.2%) (*p* = 0.047 and *p* = 0.021). On the contrary, the children from the Gp 1 of the SRS had adequacy over the recommendations for proteins higher than the children from the Gp1 of the AMS (374% vs. 343%) (*p* = 0.007).

The percentage of children with inadequate reported intakes below and above the recommendations are shown in Supplementary Table S1.

### 3.5. Contribution of Food Groups to Total Energy Intake

**Figure 1:**
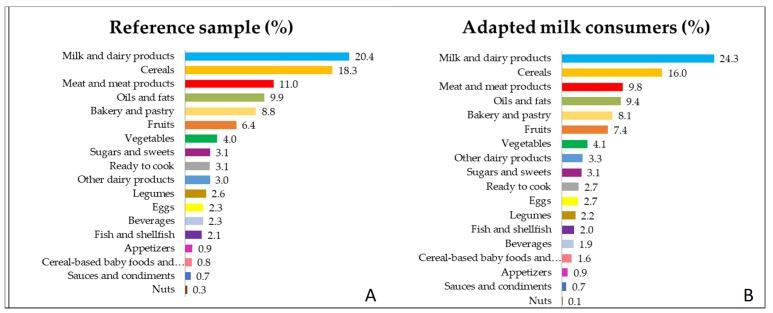
Percentage of the 18 food groups sources of the total energy intake (in %) among Spanish Pediatric Population (EsNuPI) (**A**) shows the reference sample and (**B**) the adapted milk consumers sample. There were statistically significant differences between both samples in the following food groups: Milk and dairy products, Cereals, Meat and meats products, Oils and fats, Fruits, Eggs, Beverages, Legumes, Cereal-based baby foods and Nuts (*p* < 0.005).

The contributions (%) of the 18 food groups to daily intakes of energy in the SRS and AMS are shown in Figure 1A,B, respectively and organized from the higher to the lower percentage of contribution.

In the SRS, the food groups that represent the most important sources of energy were milk and dairy products and cereals (20.4% and 18.3% from total energy intake, respectively); followed by meat and meat products (11%), oils and fats (9.86%) and bakery and pastry (8.8%), fruits (6.4%) and vegetables (4.0%). In contrast, the lowest sources of energy were the fish and shellfish (2.08%), appetizers (0.93%), cereal-based baby foods (0.81%), sauces and condiments (0.74%) and nuts (0.27%) as shown in Figure 1A.

For the AMS, the highest sources of energy, were the same as the SRS, milk and dairy products (24.3%) and cereals (16%) followed by the meat and meat products (9.77%), oils and fats (9.4%), bakery and pastry (8.09%), fruits (7.4%) and vegetables (4.1%). The lowest food group sources of energy were the fish and shellfish (1.96%), cereal-based baby foods (1.57%) appetizers (0.94%), sauces and condiments (0.69%) and nuts (0.1%) seeing that in the Figure 1B.

The following food groups contribute to a higher proportion of energy in the AMS than in the SRS—milk and dairy products (*p* < 0.001), fruits (*p* = 0.001), eggs (*p* = 0.024) and cereal-based baby foods (*p* < 0.001). Meanwhile, the food groups that contribute less to energy intake in the AMS than in the SRS were cereals (*p* < 0.001), meat and meat products (< 0.001), oils and fats (*p* = 0.044), beverages (*p* = 0.011), legumes (*p* = 0.027) and nuts (*p* = 0.002).

**Figure 2:**
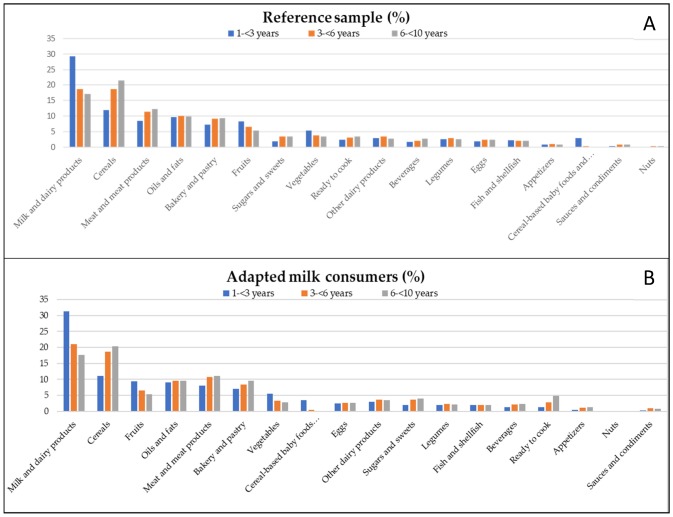
Percentage of the 18 food groups sources of the total energy intake (in %) among Spanish Pediatric Population (EsNuPI) study by age group: (**A**) shows the reference sample and (**B**) the adapted milk consumers sample.

Additionally, Figure 2A,B show the contribution (%) of the food groups categorized by age group for both samples. There were differences by groups of age between both samples (Figure 2A,B). Gp 1 of the adapted milk consumer sample had a higher contribution of energy from eggs (*p* = 0.026) and a lower energy contribution from ready to cook/eat foods (*p* = 0.006) than Gp 1 of the SRS. Gp 2 of the AMS had a higher contribution of energy from milk and dairy products (*p* = 0.002) than Gp 2 of the SRS. Gp 3 of the AMS had a higher contribution of energy from the ready to cook/eat foods (*p* = 0.009) and a lower energy contribution from vegetables (*p* = 0.001) and nuts (*p*= 0.04) than Gp 3 of the SRS.

### 3.6. Misreporting (Under- and Over-reporting of Energy Intake)

Table 6 shows the percentage of plausible reporters and non-plausible reporters of energy intake (under- and over-reporters) of the EsNuPI study.

In the SRS, the 84.7% (*n* = 598) were classified as plausible reporter and 15.3% (*n* = 108) non-plausible reporters (6.1% under- and 9.2% over-reporters). Regarding the age group, there were more under-reporters among the Gp 3 (7.6%), whereas the youngest (Gp 1) showed more over-reporters (20.5%).

The 83.5% (*n* = 618) of the AMS was classified as plausible energy reporters and 16.4% (*n* = 122) as non-plausible reporters (5.9% under- and 10.5% over-reporters). According to the age group, 10.8% (*n* = 20) were identified as under-reporters in Gp 3; whereas, 14.7% (*n* = 43) were over-reporters in Gp 1.

The data presented in this article have not been adjusted for misreporting because the exclusion of misreporters resulted in no differences in the total energy intake, so it does not significantly modify the results and conclusions of this study (Supplementary Table S2) and the distributions of relative macronutrient intakes (percentage of energy intake from proteins, carbohydrates and fat) remained almost unchanged when excluding misreports (Supplementary Tables S3 and S4).

## 4. Discussion

The EsNuPI study is the most recent survey in Spain that provides data about energy and food consumption in one general population-representative sample of milk consumers and in one sample of adapted milk consumers aged one to <10 years.

There is some controversy about the advantages and disadvantages of drinking adapted and fortified milk formulas or CM between 1–3 years old. Some studies suggest that CM is a complete and balanced food that provides high nutrient content in relation to its calorie content, so its consumption should be considered necessary from childhood to the elderly [5].

On the other hand, other studies have investigated the excessive nutrient intakes that can result from the consumption of fortified foods like the adapted and fortified milk formulas [13]. Fortification of commonly consumed foods with some micronutrients, for example, vitamin A, could result in a high percentage of young children having intakes above the tolerable upper intake level (UL) and other studies correlate the consumption of adapted and fortified milk formulas from 1–3 years old and better compliance of the intake of nutrients recommendations [10,11,14].

Understanding usual food consumption, energy and nutrient intake and nutrient adequacy to European and International standards in this specific population sample will provide useful data for identifying possible gaps and to conduct public intervention programs.

### 4.1. Total Energy Intake

Our results show that the percentage of adequacy for energy intake was 113% for the SRS and 120% for the AMS when compared to the EFSA recommendations for children aged one – <10 years [38]. When compared to the IOM recommendations for energy [39], it was 84% and 93%, respectively.

The present results are in line with other studies in Spain; for example, the ALSALMA study (2013), found an excessive energy intake, they exceeded recommended values by 135% at 7–12 months, by 123% at 13–24 months and by 124% at 25–36 months [24]. Likewise, the children (9–12 age group) (*n* = 213) from the ANIBES study (2015) covered the 82.1% of the dietary recommendation for energy according to the national currency (2013) [23]. The two abovementioned studies keep a similar dietary pattern.

The IDEFICS study with children aged 2 to 9 years found that comparing Northern (Belgium, Estonia, Germany, Sweden) and Southern European countries (Cyprus, Hungary, Italy, Spain), the total energy intake is slightly higher in Southern countries (121.5% vs. 122.8%), which is mainly due to a higher intake of proteins [18]. However, the ENALIA study, concluded that the usual intake of energy was according to the Estimated Average Requirements of the Institute of Medicine [22,39].

### 4.2. Nutrient Profile and Distribution

In our study, the mean percentage of protein contribution to the EI was 16.5% for the SRS and 15.6% for the AMS (*p* < 0.001), hence, both samples were above the upper tolerable limit of protein derived energy intake recommended by EFSA, 2015 [44] and Who, 2007 [45]; nevertheless, the AMS had adequacy closer to the recommendations and a lower percentage of protein contribution to the EI than the SRS.

Regarding to the ESPGHAN, the amount of protein in YCF available on the European market varies significantly (median 2.6 g/100 kcal); hence, the majority of the YCF has a lower protein content than the regular cow´s milk (4.8 g/100 kcal). Consequently, children who consume YCF, that is, SMA have a lower intake of protein when compared to the children that majorly consume cow´s milk, that is, SRS [11].

In accordance with the EFSA, the dietary reference intake for total protein is about 0.90 to 1.14 g/kg body weight for children. As we have data on self-reported weight for our study population, we were able to analyze the adequacy of protein intake that was 3.5 times higher than the EFSA and 3.4 times than the IOM recommendations [38,39].

Increasing research suggests that high protein intake may be detrimental to metabolic risk factors such as for overweight in infants and toddlers [46,47,48] by increasing body weight in the absence of improvements in fat-free mass [49]. It is believed that protein consumption stimulates the secretion of insulin-like growth factor I, which leads to cellular proliferation, accelerated growth and increased body fat [50,51].

The latest Spanish data on infants and young children are described in the ENALIA, ALSALMA and ANIBES studies. In all cases, the reported protein intake was also higher than the available recommendations [24,52,53].

The ENALIA study concluded that protein contribution to energy was 16.7% for children aged 1–3 years old and 17.1% for children 4–8 years old, when using the Acceptable Macronutrient Distribution Range (AMDR) [22]. Besides, in the ALSALMA study, 95.9% of the children between 7 and 36 months had a protein consumption more than twice the Recommended Daily Allowances (RDA) [24]. Finally, when comparing our data with the ANIBES study, the results are similar, overall energy intake derived from protein was 16.8%. Only 10% of the ANIBES population was within the recommended range for protein intake (<15%) [23].

Similar results have been found in other European countries for this age group. Huysentruyt et al. found that all the Belgium children between 6 to 36 months (*n* = 500) had a protein intake above the recommended dietary intakes and for 156 children (33.5%), were above the UL of 15% of total energy intake that was proposed by ESPGHAN [54,55]

The NUTRINTAKE cross-sectional study (*n* = 390) in Italian children aged 6 months to 3 years found a high intake of proteins, simple carbohydrates, saturated fats and sodium compared with the Italian recommended dietary allowances [56].

In 2012, the Estudo do Padrão Alimentar e de Crescimento Infantil (EPACI) a cross-sectional study (*n* = 2232) in Portuguese children aged 12–36 months, found that the mean daily protein intake was four times more than the RDA [57].

Moreover, Brunner et al. found that in Swiss toddlers between one to 3 years (*n* = 188) the protein intake was three to fourfold higher than the recommended daily intake (RDI) and reached the upper limit of 15% of total energy intake [27].

Regarding carbohydrates, the EFSA recommendations (38) for children, specify that this macronutrient should provide 45%–60% of the energy intake; and the IOM recommends an intake of 45%–65% of the total energy. In the present study, the percentage of carbohydrates contribution to the energy intake was 45.4% for the SRS and 46.7% for the AMS; hence, both samples mean values were in accordance with the recommendations and were in line with the findings of other studies [22,58,59]. Nevertheless, the percentage of children that were below the EFSA and the IOM recommendations was 47.8% in the SRS and 39.3% in the AMS (*p* < 0.001) [38,39].

Consistently with our results, in the ENALIA study, the median values from carbohydrates were 46.8% of total energy and the proportion of participants below the lower limit of the AMDR was between 35.7–28.7% for boys and 42.9–29.7% for girls [22]. In the ALSALMA study, a low energy intake of 41.1% from carbohydrates was observed according to the RDA [24]. In the ANIBES study, a low contribution from carbohydrates to energy intake was found (41.4%) according to the EFSA recommendations [23].

In European countries, data from dietary surveys show that average carbohydrates intakes for children and adolescents ranged between 43% and 58% of the total energy intake [59,60]. The GENESIS cross-sectional study (*n* = 2374) in Greek children aged one to 5 years concluded that 21.0% of the children had lower intakes for carbohydrates, according to the AMDR [61]. On the contrary, in the IDEFICS study, the mean percentage of usual energy intake from carbohydrates was 52.1%; thus, according to this study, the majority of European children were complying with common macronutrient intake recommendations [18].

The EFSA suggests consuming 35–40% energy intake from fat for children one – <3 years old and 20–35% for children 4–17 years old and according to the IOM recommendations range 30% to 40% for toddlers between one to 3 years and 25–35% for children between 4 to <10 years of energy from total fat [38].

The percentage of fat contribution to the energy intake was 36.5% for the SRS and 35.9% for the AMS; consequently, the percentage of fat was higher than the EFSA and IOM recommendations. Additionally, in both samples of our study, there was a high percentage of children above the EFSA and the IOM recommendations for fat; being SRS the sample with the higher percentage of children above both recommendations (47.2% for EFSA and 38.5% for IOM, *p* < 0.001)

Similarly, in the ANIBES study, the total fat intake was higher (37.9%) than the EFSA recommendations [53]. Moreover, a systematic review conducted by Wanden-Berghe et al., in 2015 in Spanish population 5 to 81 years old, found that the mean energy contribution from macronutrients was 18% protein, 44% carbohydrates and 38% fat. Therefore, the Spanish population of this review does not meet the macronutrient intake recommendations [62].

Manios et al., in the GENESIS study, concluded that the “at risk of being overweight” and the “overweight” children consumed more total energy, protein and fat compared with their normal-weight counterparts [61]. However, the IDEFICS study found that only 25% of the European children consume more fat than the recommended in the D-A-C-H references values (nutritional guidelines for Germany, Switzerland and Austria) [18].

In general, in our study, we can observe that both samples do not meet the EFSA and the IOM recommendations for the protein and fat distribution range (high consumption of protein and fat) and adequate consumption of carbohydrates as a whole; nevertheless, a high percentage of children do not meet the recommendations. Likewise, according to the EFSA, intervention studies concluded that high fat (>35% E) and low carbohydrates (<50% E) diets are associated with adverse short- and long-term effects on body weight [52]. Similar results were found in two groups of Irish children (*n* = 85) ages 12-24 months that consumed growing up milks (GUM) together with cow’s milk (*n* = 29) or cow’s milk only (*n* = 56). While average total daily energy intakes were similar in both consumers and non-consumers of GUM (1050 kcal vs 1027 kcal), intakes of protein (14.3% vs. 17%) and fat (33.2% vs. 35.1%) were lower and intakes of carbohydrate were higher in consumers of GUM (48.4% vs. 44.6%) [63].

The high protein and fat intakes in Spanish children represent a major concern. In Spain, 40% of children have overweight or obesity and there is an association between high protein intakes, adiposity and a higher body mass index (BMI) in infancy and early childhood [22,25]. Moreover, the high percentage of subjects that were above the fat recommendations is worrying and should be modified. High-fat diets may decrease insulin-sensitivity and are positively associated with changes in fasting and postprandial factor VII, which may increase cardiovascular risk and other chronic diseases [39]. Nevertheless, it is crucial to maintain an adequate intake of carbohydrates, because they are essential to the contribution of energy and glucose to the brain, which is the only carbohydrates-dependent organ in the body [40]. Likewise, high carbohydrates diets tend to induce adverse effects on the blood lipid profile [58].

### 4.3. Contribution of Food Groups to Energy Intake

In this study, the contribution of food groups to energy in Spanish children one to <10 years old is reported. To our knowledge, this is the first study that provides this information of Spanish children in this range of age.

In both samples, the milk and dairy products were the major contributors to the total energy among all 18 food groups, providing a series of critical nutrients but the contribution was significantly higher in the AMS (24.3%) than in the SRS (20.4%) (*p* < 0.001). Overall, in infancy, the adapted and fortified milk formulas are important contributors to the total energy and nutrient intakes beyond calcium, such as potassium and vitamin D [11,64].

Furthermore, it is important to emphasize that the consumption of milk and dairy products promote adherence to the nutrient recommendations and may protect against the most prevalent non-communicable chronic diseases [8,9]. Similarly, in the NHANES study, found that milk was the top food source of energy in all ages [32].

In both study samples, the cereals food group followed the milk and dairy products as the second contributor to energy intake. Cereals are the main staple food in many diets, providing a large percentage of daily energy intake. In a balanced diet, cereals represent a healthy source of multiple nutrients, dietary fibers and bioactive peptides with anticancer, antioxidant and antithrombotic effects [65]. This food group represents the first contributor to energy intake in the ANIBES study population aged 9 to 17 years [23].

Additionally, our results show that meat and meat products are the third contributor to energy intake in both samples; this increased in accordance with the age. However, the percentage of contribution to energy from meat and meat products was significantly higher in the SRS than in the AMS (11% vs. 7.8%) (*p* < 0.001). As informed by EFSA in 2012 [58], in most European countries, the main contributors to the dietary protein intake are meat and meat products. In the Helena study, conducted in 2006–2007 with children aged 12.5–17.5 years, the average total protein intake exceeded the recommendations of the WHO and EFSA and 59% of total protein intake was derived from animal protein [66].

Meat contributes to important nutrients such as protein, saturated fatty acids, vitamins B, vitamin D and essential minerals, for example, iron (Fe) and zinc (Zn); however, processed meat is the principal food that contributes to SFA and sodium intakes across all ages and stages of life [39,58,67]. Additionally, a high SFA intake has been identified as a risk factor for weight gain or higher weight; this has been associated with an increased cardiovascular risk [68,69].

Similarly, with our results, in the Melbourne Infant Feeding Activity and Nutrition Trial (InFANT) (*n* = 542) developed in Australian children aged 9 months to 5 years, the principal sources of energy intake were milk and dairy products, cereals and bread and meat-meat products [70].

The fourth group contributing to energy intake was oils and fats but there were no differences by groups between the two samples. Similar to our results, in the ANIBES study, the food group of oils and fats represents the third contributor to energy intake and the group of children and adolescents had a lower percentage of contribution of oils and fats than the adults (10% EI and 15% EI, respectively) [23].

Furthermore, in our study population, there is a consistent trend toward a decrease in the consumption of fruit and vegetables as age increases, indicating that they were not sufficiently consumed. Several studies have shown that the consumption of these foods groups protects against chronic diseases and obesity because their high level of water and fiber, so incorporating them into the diet can reduce energy density, promote satiety and decrease energy intake [71,72]. Similarly, a cross-sectional study on a sample of GENESIS cohort of 2287 children (aged one to 5 years) concluded that about 80% of the children had a poor diet associated with low fruit, vegetable and grains intake and high saturated fat intake [61]. Moreover, in the NHANES study, for children 2–5 and 6–11 years old, the fruit and vegetables were the 7º and 16º ranked food sources of energy, respectively [32].

Finally, the contribution of bakery, sugar and sweets and ready to cook/eat products to total energy intake augmented with increasing age in SRS and AMS. The consumption of sugar and sweets and bakery is of concern because they are rich in free sugars and according to the World Health Organization their intake should be limited to no more than 10% of the total energy and preferably to no more than 5% in children [73]. Additionally, high sugar consumption increases the risk for overweight, obesity and dental caries, which can result in poor nutrient supply and reduced dietary diversity and may be associated with increased risk of type 2 diabetes mellitus, cardiovascular risk and other health effects [74].

In general, it can be observed that the SRS consumed significantly more cereals, meat and meat products, oils and fats, beverages, legumes and nuts than the AMS. Meanwhile, the AMS has a significantly higher consumption of milk and dairy products, fruits, eggs and cereal-based baby foods and supplements. Likewise, Ortega et al. studied the association between dairy consumption and dietary patterns and intake of selected nutrients in 7–11-year-old children and concluded that children who drank more milk also had better dietary patterns [9]. Santaliestra-Pasías et al. concluded that the consumption of dairy products could be part of a healthy lifestyle, including physical activity and low time of sedentary behavior activities [8].

### 4.4. Energy Misreporting

The exclusion of over- and under-reporters from our analyses did not significantly modify the results of our study. Misreporting represent 15.3% and 16.4% for our SRS and AMS, respectively; this is lower than in other national [22,75] and European studies [18,28,76]. However, in accordance with the EFSA recommendations, we did not exclude potential misreporters from the analyses [31].

According to age groups, over-reporting was higher in group 1) 1–<3 years old (20.5% SRS; 14.7% adapted milk consumers). Similarly, Burrows et al. (2010) [77] conducted a systematic review of studies which assessed the validity of energy intakes reported using a variety of dietary assessment methods with children from birth to 18 years, in comparison to double-labelled water (DLW). They found that for 24-h DR, over-reporting was most common in this age group.

Conversely, the Gp 3 had more children classified as under-reporters. Livingstone and Black (2003) [78] reviewed studies where children’s reported energy intake had been compared with energy expenditure measured by DLW and they found a trend for under-reporting to increase with age.

### 4.5. Strengths and Limitations

The major strength of the present study was the possibility of examining a representative sample of the Spanish children aged one to <10 years living in urban areas and the possibility of comparing the results of this reference population with a sample of adapted milk consumers of the same age. Moreover, special care was taken in the design, protocol and methodology of the study to ensure that sampling was carried out very carefully. Consequently, the 24-h DR information was collected following the methodology recommended by EFSA (The PAN CAKE-Pilot study) [33,37].

When children are the subjects of dietary assessment, the challenges are increased due to limited literacy, writing skills, food knowledge and often interest in taking part in dietary surveys coupled with the range of people responsible for their care and food provision [78]. However, in our study, the percentages of misreporting were low compared with other national and international studies [18,22,28,75]. Misreporting of dietary intake is a major issue in dietary recall methods as the 24-h DR [21]. To mitigate this limitation, the Goldberg cut-offs method to identify the misreporters according to the EFSA recommendations was used in our study [31]. Likewise, the interviewers were previously trained by experiences dieticians/nutritionists; they were professionals of different areas and degrees (e.g., law, history, nursing and designer degrees, administrative work, etc.). The dieticians/nutritionist of the entities in charge of the study were responsible for checking the food consumption records during the study process and from the coding process to verify the survey’s information. Besides, the children’s weight and height were reported by their parents using the health’s card of children, which could have generated a small gap in the calculation of the protein intake recommendations.

## 5. Conclusions

Spanish children aged one to <10 years old of the EsNuPI study reported adequate but somewhat high, energy intake and are in accordance with other studies subjects of the comparable age group. Energy distribution follows the trend, like other studies, of high protein and fat contributions to total energy intake. Also, a great percentage of children were below the lower limit of the recommendations for carbohydrates. Regarding the food groups contribution to energy, the SRS reported more contribution to energy from cereals, meat and meat products, bakery and pastry and ready to cook/eat foods than the AMS; meanwhile, the AMS reported more energy from milk and dairy products, fruits and eggs than the SRS. Those results suggest that the children consuming adapted milk have better adherence to the food-based dietary guidelines. More analyses are warranted in order to characterize food patterns and confirm the quality of the diet in our study population.

## Figures and Tables

**Table 1:** Calculated energy cut-off for at group and individual levels for the Nutritional Study in Spanish Pediatric Population (EsNuPI) (*n* = 1448).

		Misreporting Cut-Off			
		Group Level		Individual Level	
	PAL	Lower	Upper	Lower	Upper
**Reference sample**	1.57	1.54	1.60	0.98	2.52
1 to <3 years	1.57	1.51	1.62	0.97	2.51
3 to <6 years	1.56	1.51	1.61	0.97	2.50
6 to <10 years	1.58	1.54	1.63	0.99	2.54
**Adapted milk consumers sample**	1.55	1.52	1.57	0.96	2.49
1 to <3 years	1.54	1.50	1.58	0.96	2.47
3 to <6 years	1.52	1.48	1.57	0.95	2.45
6 to <10 years	1.61	1.55	1.66	1.00	2.58

PAL: Physical activity level. The PAL was calculated for individual and group level according to the European Food Safety Authority (EFSA) protocol to assess misreporting [31].

**Table 2:** Distribution of the studied sample in the Nutritional Study in Spanish Pediatric Population (EsNuPI) (*n* = 1448).

		Whole Population	Reference Sample	Adapted Milk Consumers
		*n* = 1448	*n* = 707	*n =* 741
Sex	Boys	728	357	371
	Girls	720	350	370
Age (years)	1 to <3	456	162	294
	3 to <6	506	244	262
	6 to <10	486	301	185

Distribution of the studied sample in the EsNuPI study within subjects with complete data of two 24-h Dietary recall as well as complete information on the variables of interest.

**Table 3:** Reported energy intake for the Nutritional Study in Spanish Pediatric Population (EsNuPI) according to age group and sex (*n* = 1448).

	Reference Sample																			
Energy (kcal/day)	Total					1–<3 years					3–<6 years					6–<10 years				
	n	Mean	SD	Median	IQR	n	Mean	SD	Median	IQR	n	Mean	SD	Median	IQR	n	Mean	SD	Median	IQR
Total	707	1503	417	**1484**	526	162	1229	347	1215^a^	485	244	1492	347	1497^b^	431	30	1660	427	1600^c^	533
Boys	357	1534	427	**1515**	491	84	1238	346	1202^a^	522	122	1509	360	1502^b^	380	151	1718	423	1661^c^	562
Girls	350	1472	405	**1461**	539	78	1220	350	1219^a^	435	122	1475	333	1477^b^	515	150	1601	424	1538^b^	510
	**Adapted Milk Consumers Sample**																			
**Energy (kcal/day)**	**Total**					**1–<3 years**					**3–<6 years**					**6–<10 years**				
	***n***	**Mean**	**SD**	**Median**	**IQR**	***n***	**Mean**	**SD**	**Median**	**IQR**	***n***	**Mean**	**SD**	**Median**	**IQR**	***n***	**Mean**	**SD**	**Median**	**IQR**
Total	741	1404	394	**1375**	491	294	1181	306	1163^a^	375	262	1497	371	1475^b^	456	185	1626	375	1577^c^	464
Boys	371	1405	409	**1367**	485	144	1171	305	1165^a^	402	128	1478	399	1473^b^	429	99	1652	377	1593^c^	476
Girls	370	1402	379	**1376**	500	150	1191	307	1162^a^	392	134	1514	343	1481^b^	496	86	1596	373	1553^b^	456

Average energy intake values for two 24-h dietary recalls were used. Results are expressed as the mean, standard deviation, median and interquartile range (IQR). Mann-Whitney U test was used to evaluate differences by sex and age groups between the reference and adapted milk consumers sample (median values, significant differences are shown in bold type). Kruskal-Wallis test was used to calculate differences among age groups within samples (median values, values with different superscript letters were significantly different). *p*-value <0.05 was considered statistically significant.

**Table 4:** Distribution of reported intakes of macronutrients as a percentage of the total energy intake (%EI) from two 24-h dietary recalls from the Nutritional Study in Spanish Pediatric Population (EsNuPI) according to sex and age group (*n* = 1448).

	Reference Sample									Adapted Milk Consumers								
	Total		1–<3 years		3–<6 years		6–<10 years			Total		1–<3 years		3–<6 years		6–<10 years		
	*n*	*%*	*n*	%	*n*	%	*n*	%	*p*	*n*	%	*n*	%	*n*	%	*n*	%	*p*
(%) Proteins	707	**16.5**	162	**15.9** ^a^	244	**16.8** ^b^	301	16.6 ^b^	0.009	741	**15.6**	294	**15.0** ^a^	262	**15.9** ^b^	185	16.2 ^b^	<0.001
Boys	357	**16.4**	84	15.9	122	16.7	151	16.5	0.104	371	**15.5**	144	14.7 ^a^	128	15.9 ^b^	99	16.3 ^b^	<0.001
Girls	350	**16.6**	78	16.0	122	16.9	150	16.8	0.082	370	**15.7**	150	15.4	134	15.9	86	16.1	0.143
(%) Carbohydrates	707	**45.4**	162	**46.2**	244	45.2	301	45.1	0.176	741	**46.7**	294	**48.3** ^a^	262	45.9 ^b^	185	45.3 ^b^	<0.001
Boys	357	**45.6**	84	46.2	122	45.6	151	45.1	0.40	371	**46.8**	144	48.3 ^a^	128	46.0 ^b^	99	45.4 ^b^	<0.001
Girls	350	**45.2**	78	46.2	122	44.8	150	45.1	0.33	370	**46.7**	150	48.3 ^a^	134	45.8 ^b^	86	45.2 ^b^	<0.001
(%) Fat	707	36.5	162	**36.2**	244	36.4	301	36.7	0.63	741	35.9	294	**34.6** ^a^	262	36.6 ^b^	185	37.0 ^b^	<0.001
Boys	357	36.4	84	36.2	122	36.0	151	36.9	0.49	371	36.0	144	35.1	128	36.5	99	36.8	0.056
Girls	350	36.6	78	36.2	122	36.8	150	36.6	0.79	370	35.8	150	34.2 ^a^	134	36.8 ^b^	86	37.2 ^b^	<0.001

EI: Energy intake. Results are expressed in percentage of contribution to the total energy intake. *t*-test was used to evaluate differences by sex and age groups between the reference and adapted milk consumers sample (mean values, significant differences are shown in bold type). ANOVA analysis was used to calculate differences among age groups (mean values; values with different superscript letters were significantly different). *p*-value <0.05 was considered statistically significant.

**Table 5:** Adequacy to the European Food Safe Authority and Institute of Medicine recommendations for energy and protein daily intakes and percentages of children that meet these criteria for fat and carbohydrates by sample, age group and sex for the Nutritional Study in Spanish Pediatric Population (EsNuPI) (*n* = 1448).

	Reference Sample									Adapted Milk Consumers								
	Total		1–<3 years		3–<6 years		6–<10 years			Total		1–<3 years		3–<6 years		6–<10 years		
	*n*	%	*n*	%	*N*	%	*n*	%	*p*	*n*	%	*n*	%	*n*	%	*n*	%	*p*
**Adequacy to recommendations** ^1^																		
(%) Energy intake EFSA	707	**113**	162	141 ^a^	244	115 ^b^	301	96.8 ^c^	<0.001	741	**120**	294	139 ^a^	262	117 ^b^	185	94.9 ^c^	<0.001
Boys	357	111	84	133 ^a^	122	113 ^b^	151	97.3 ^c^	<0.001	371	114	144	132 ^a^	128	111 ^b^	99	93.2 ^c^	<0.001
Girls	350	**116**	78	150 ^a^	122	118 ^b^	150	96.4 ^c^	<0.001	370	**126**	150	145 ^a^	134	123 ^b^	86	96.9 ^c^	<0.001
(%) Energy intake IOM	707	**84.0**	162	127 ^a^	244	75.6 ^b^	301	67.6 ^c^	<0.001	741	**93.4**	294	125 ^a^	262	78.0 ^b^	185	67.4 ^c^	<0.001
Boys	357	**80.0**	84	122 ^a^	122	70.8 ^b^	151	64.0 ^c^	<0.001	371	**89.2**	144	123 ^a^	128	71.8 ^b^	99	62.8 ^c^	<0.001
Girls	350	**88.0**	78	132 ^a^	122	80.3 ^b^	150	71.3 ^c^	<0.001	370	**98.4**	150	126 ^a^	134	83.9 ^b^	86	72.8 ^c^	<0.001
(%) Proteins EFSA	707	353	162	**400** ^a^	244	414 ^a^	301	278 ^b^	<0.001	741	362	294	**371** ^a^	262	411 ^b^	185	278 ^c^	<0.001
Boys	357	351	84	391 ^a^	122	411 ^a^	151	281 ^b^	<0.001	371	350	144	358 ^a^	128	400 ^b^	99	275 ^c^	<0.001
Girls	350	355	78	411 ^a^	122	417 ^a^	150	275 ^b^	<0.001	370	374	150	385 ^a^	134	422 ^b^	86	281 ^c^	<0.001
(%) Proteins IOM	707	351	162	**374** ^a^	244	385 ^a^	301	312 ^b^	<0.001	741	342	294	**343** ^a^	262	369 ^b^	185	305 ^c^	<0.001
Boys	357	**358**	84	377 ^a^	122	388 ^a^	151	324 ^b^	<0.001	371	**335**	144	333 ^a^	128	359 ^a^	99	306 ^b^	<0.001
Girls	350	345	78	371 ^a^	122	381 ^a^	150	301 ^b^	<0.001	370	350	150	352 ^a^	134	378 ^a^	86	303 ^b^	<0.001
**Children meeting the recommendations** ^2^																		
(%) Carbohydrates EFSA	707	**51.1**	162	58.0	244	47.1	301	50.5	0.21	741	**58.6**	294	65.0 ^a^	262	56.5 ^ab^	185	51.4 ^c^	<0.001
Boys	357	**51.5**	84	60.7	122	46.7	151	50.3	0.053	371	**58.5**	144	66.0 ^a^	128	57.0 ^ab^	99	49.5 ^b^	0.018
Girls	350	50.6	78	55.1	122	47.5	150	50.7	0.66	370	58.6	150	64.0	134	56.0	86	53.5	0.098
(%) Carbohydrates IOM	707	**51.9**	162	**58.6**	244	48.4	301	51.2	0.20	741	**60.6**	294	**68.7** ^a^	262	57.6 ^b^	185	51.9 ^b^	0.002
Boys	357	52.1	84	60.7	122	48.4	151	50.3	0.27	371	60.6	144	70.1 ^a^	128	57.8 ^ab^	99	50.5 ^a^	0.018
Girls	350	**51.7**	78	56.4	122	48.4	150	52.0	0.29	370	**60.5**	150	67.3	134	57.5	86	53.5	0.074
(%) Fat EFSA	707	**37.1**	162	**27.2** ^a^	244	39.3 ^b^	301	40.5 ^b^	<0.001	741	**34.0**	294	**29.9**	262	36.6	185	63.2	<0.001
Boys	357	**37.3**	84	27.4	122	41.0	151	60.3	<0.001	371	**38.0**	144	34.0	128	39.1	99	57.6	<0.001
Girls	350	**36.9**	78	26.9	122	37.7	150	41.3	<0.001	370	**30.0**	150	26.0	134	34.3	86	30.2	<0.001
(%) Fat IOM	707	**45.4**	162	**53.1** ^a^	244	48.0 ^ab^	301	39.2 ^b^	<0.001	741	**49.1**	294	**58.8** ^a^	262	47.7 ^b^	185	35.7 ^c^	<0.001
Boys	357	**46.2**	84	52.4	122	50.8	151	39.1	<0.001	371	**50.9**	144	58.3 ^a^	128	50.0 ^ab^	99	41.4 ^b^	<0.001
Girls	350	**44.6**	78	53.8	122	45.1	150	39.3	<0.001	370	**47.3**	150	59.3 ^a^	134	45.5 ^a^	86	29.1 ^b^	<0.001

Results are expressed in percentage (%). Recommended daily intakes, according to Europe Food Safety Authority (EFSA) and Institute of Medicine (IOM) [38,39]. ^1^ *t*-test was used to evaluate differences for the adequacy to the EFSA and IOM recommendations for energy and protein intakes by sex and age groups between the reference and adapted milk consumers sample (mean values, are shown in boldface type). ANOVA analysis was used to calculate differences for the adequacy to the EFSA and IOM recommendations for energy and protein intakes among age groups (mean values; values with different superscript letters were significantly different). ^2^ *Chi*-squared test was used to evaluate differences within the percentage of samples that meet these criteria for fat and carbohydrates recommendations by sex and age groups between the reference and adapted milk consumers sample (mean values, are shown in bold type). *Z*-test was used to calculate differences within the percentage of samples that meet these criteria for fat and carbohydrates recommendations among age groups (mean values; values with different superscript letters were significantly different).

**Table 6:** Percentages of under-reporters, plausible and over-reporters of energy intake in the Nutritional Study in Spanish Pediatric Population (EsNuPI) (number and percentages) (*n* = 1446) *.

	Reference Sample (*n* = 706)									Adapted Milk Consumers (*n* = 740)								
	Total		1–<3 years		3–<6 years		6–<10 years			Total		1–<3 years		3–<6 years		6–<10 years		
	*n*	%	*n*	%	*n*	%	*n*	%	*p*	*n*	%	*n*	%	*n*	%	*n*	%	*p*
Plausible reporters	598	84.7	120	**74.5**	211	86.5	267	**88.7**	<0.001	618	83.5	236	**80.5**	224	85.5	158	**85.4**	<0.001
Non-Plausible reporters	108	15.3	41	25.5	33	13.5	34	11.3	<0.001	122	16.4	57	19.5	38	14.5	27	14.6	<0.001
Under-reporters	43	6.1	8	5.0	12	4.9	23	7.6	0.015	44	5.9	14	4.8	10	3.8	20	10.8	0.178
Over-reporters	65	9.2	33	20.5	21	8.6	11	3.7	0.004	78	10.5	43	14.7	28	10.7	7	3.8	<0.001

Results are expressed in percentage of the total population that is plausible, under and over-reporters (%). Chi-squared test was used to evaluate differences between the reference and adapted milk consumers sample (mean values, significant differences are shown in bold type) and to calculate differences among age groups. *Data from a total sample of 1446 participants of the EsNuPI study have been used for the evaluation of misreporting, due to the lack of information on physical activity data of two study participants (*n* = 1 SRS; *n* = 1 AMS).

## Supplementary Materials

The following are available online at https://www.mdpi.com/2072-6643/12/4/893/s1, Table S1: The inadequacy to the European Food Safe Authority and Institute of Medicine recommendations for percentages of samples that do not meet these criteria for fat and carbohydrates by age group and sex in the Spanish Pediatric Population (EsNuPI) study (*n* = 1448), Table S2: Reported energy intake for the total and for the plausible reporters of the Nutritional Study in Spanish Pediatric Population (EsNuPI) according to age group and sex (*n* = 1446) *; Table S3: Distribution of reported intakes of macronutrients as percentage of the total energy intake (%EI) from two 24-h dietary recalls for the total sample and for the plausible reporters of the Nutritional Study in Spanish Pediatric Population (EsNuPI) according to sex and age group (*n* = 1446) *; Table S4: The adequacy to the European Food Safe Authority and Institute of Medicine recommendations for energy and protein intakes and percentages of samples that meet these criteria for fat and carbohydrates for the plausible sample by age group and sex for the Nutritional Study in Spanish Pediatric Population (EsNuPI) (*n* = 1448).
