# Supplementary material for: Energy Intake, Macronutrient Profile and Food Sources of Spanish Children Aged One to <10 Years—Results from the EsNuPI Study
Source: Nutrients. 2020 Mar 25;12(4):893. doi: 10.3390/nu12040893 (PMC7231217; doi:10.3390/nu12040893)
Supplement: Supplementary file 1 [file nutrients-12-00893-s001.pdf]

Supplementary Table 1. The inadequacy to the European Food Safe Authority and Institute of Medicine recommendations for percentages of samples that do not meet these criteria for fat and carbohydrates by age group and sex in the Spanish Pediatric Population (EsNuPI) study (*n* = 1448).

	Reference sample												Adapted milk consumers											
	Total			1-<3 years			3-<6 years			6-<10 years			Total			1-<3 years			3-<6 years			6-<10 years		
	n	% BR	% AR	n	% BR	% AR	N	% BR	% AR	n	% BR	% AR	n	% BR	% AR	n	% BR	% AR	n	% BR	% AR	n	% BR	% AR
Subjects not meeting the recommendations [1]																								
**(%) Carbohydrates EFSA**	707	47.8	1.1	162	40.7	1.2	244	51.2	1.6	301	48.8	0.7	741	**39.3**	**2.2**	294	31.3 ^{a}	3.7 ^{a}	262	42.0 ^{b}	1.5 ^{a}	185	48.1 ^{b}	0.5 ^{a}
Boys	357	**47.6**	**0.8**	84	39.3	0.0	122	50.8	2.5	151	49.7	0.0	371	**39.1**	**2.4**	144	29.9 ^{a}	4.2 ^{a}	128	41.4 ^{ab}	1.6 ^{a}	99	49.5 ^{b}	1.0 ^{a}
Girls	350	48.0	1.4	78	42.3	2.6	122	51.6	0.8	150	48.0	1.3	370	39.5	1.9	150	32.7	3.3	134	42.5	1.5	86	46.5	0.0
**(%) Carbohydrates IOM**	707	47.8	0.3	162	**40.7**	**0.6**	244	51.2	0.4	301	48.8	0.0	741	**39.3**	**0.1**	294	**31.3** ^{a}	**0.0** ^{a}	262	42.0 ^{b}	0.4 ^{a}	185	48.1 ^{b}	0.0 ^{a}
Boys	357	47.6	0.3	84	39.3	0.0	122	50.8	0.8	151	49.7	0.0	371	39.1	0.3	144	29.9 ^{a}	0.0 ^{a}	128	41.4 ^{ab}	0.8 ^{a}	99	49.5 ^{b}	0.0 ^{a}
Girls	350	**48.0**	**0.3**	78	42.3	1.3	122	51.6	0.0	150	48.0	0.0	370	**39.5**	**0.0**	150	32.7	0.0	134	42.5	0.0	86	46.5	0.0
**(%) Fat EFSA**	707	**15.7**	**47.2**	162	**43.8** ^{a}	**29.0** ^{a}	244	16.0 ^{b}	44.7 ^{b}	301	0.3 ^{c}	59.1 ^{c}	741	**27.5**	**38.5**	294	**52.0** ^{a}	**18.0** ^{a}	262	19.5 ^{b}	43.9 ^{b}	185	0.0 ^{c}	63.2 ^{c}
Boys	357	**16.8**	**45.9**	84	45.2 ^{a}	27.4 ^{a}	122	18.0 ^{b}	41.0 ^{a}	151	0.0 ^{c}	60.3 ^{b}	371	**24.3**	**37.7**	144	46.5 ^{a}	19.4 ^{a}	128	18.0 ^{b}	43.0 ^{b}	99	0.0 ^{c}	57.6 ^{b}
Girls	350	**14.6**	**48.6**	78	42.3 ^{a}	30.8 ^{a}	122	13.9 ^{b}	48.4 ^{b}	150	0.7 ^{c}	58.0 ^{b}	370	**30.8**	**39.2**	150	57.3 ^{a}	16.7 ^{a}	134	20.9 ^{b}	44.8 ^{b}	86	1.2 ^{c}	69.8 ^{c}
**(%) Fat IOM** [2]	707	**7.4**	**47.2**	162	**17.9** ^{a}	**29.0** ^{a}	244	7.4 ^{b}	44.7 ^{b}	301	1.7 ^{c}	59.1 ^{c}	741	**12.4**	**38.5**	294	**23.1** ^{a}	**18.0** ^{a}	262	8.4 ^{b}	43.9 ^{b}	185	1.1 ^{c}	63.2 ^{c}
Boys	357	**7.8**	**45.9**	84	20.2 ^{a}	27.4 ^{a}	122	8.2 ^{b}	41.0 ^{a}	151	0.7 ^{c}	60.3 ^{b}	371	**11.3**	**37.7**	144	22.2 ^{a}	19.4 ^{a}	128	7.0 ^{b}	43.0 ^{b}	99	1.0 ^{b}	57.6 ^{b}
Girls	350	**6.9**	**48.6**	78	15.4 ^{a}	30.8 ^{a}	122	6.6 ^{ab}	48.4 ^{b}	150	2.7 ^{b}	58.0 ^{b}	370	**13.5**	**39.2**	150	24.0 ^{a}	16.7 ^{a}	134	9.7 ^{b}	44.8 ^{b}	86	1.2 ^{c}	69.8 ^{c}

BR: below the recommendations; AR: above recommendations. Results are expressed in percentage. Recommended daily intakes according to Europe Food Safety Authority (EFSA) and for Americans, Institute of Medicine (IOM) [31,32]. Percentage for inadequacy was calculated comparing with of the EFSA and IOM recommendations. In the first column percentage below the recommendations and in the second percentage above the recommendations. [1] *Chi* squared test were used to evaluate differences within the percentage of samples that do not meet these criteria for fat and carbohydrates recommendations by sex and age groups between the reference and adapted milk consumers sample (mean values, are shown in bold type). *Z*-test was used to calculate differences within the percentage of samples that do not meet these criteria for fat and carbohydrates recommendations among age groups (mean values; values with different superscript letters were significantly different).

Supplementary Table 2. Reported energy intake for the total and for the plausible reporters of the Nutritional Study in Spanish Pediatric Population (EsNuPI) according to age group and sex (*n* = 1446) *.

	Total and plausible reporters																				
**Energy (Kcal/day)**	**Total**					**1-<3 years**					**3-<6 years**					**6-<10 years**					
	n	**Mean**	**SD**	**Median**	**IQR**	***n***	**Mean**	**SD**	**Median**	**IQR**	***n***	**Mean**	**SD**	**Median**	**IQR**	***n***	**Mean**	**SD**	**Median**	**IQR**	***P***
Reference	707	1503	417	1484	526	162	1229	347	1215 ^{a}	485	244	1492	347	1497 ^{b}	431	30	1660	427	1600 ^{c}	533	<0.001
Reference plausible	598	1507	386	1491	488	120	1169	292	1162 ^{a}	389	211	1491	300	1480 ^{b}	369	267	1672	382	1624 ^{c}	479	<0.001
Adapted milk consumers	741	1404	394	1375	491	294	1181	306	1163 ^{a}	375	262	1497	371	1475 ^{b}	456	185	1626	375	1577 ^{c}	464	<0.001
Adapted milk consumers plausible	618	1391	354	1365	460	236	1148	234	1149 ^{a}	326	224	1456	295	1471 ^{b}	405	158	1662	341	1602 ^{c}	408	<0.001

Average energy intake values for two 24-h dietary recalls were used. Results are expressed as the mean, standard deviation, median and interquartile range (IQR) Kruskal-Wallis analysis was used to calculate differences among age groups (median values, values with different superscript letters were significantly different). P-value <0.05 was considered statistically significant. * Data from a total sample of 1446 participants of the EsNuPI study have been used for the evaluation of misreporting, due to the lack of information on physical activity data of two study participants (*n* = 1 SRS; *n* = 1 AMS).

Supplementary Table 3. Distribution of reported intakes of macronutrients as percentage of the total energy intake (%EI) from two 24-h dietary recalls for the total sample and for the plausible reporters of the Nutritional Study in Spanish Pediatric Population (EsNuPI) according to sex and age group (*n* = 1446) *.

	Total		1-<3 years		3-<6 years		6-<10 years		
**(%) Proteins**	*n*	%	*n*	%	*n*	%	*n*	%	***P***
Reference	707	**16.5**	162	15,9 [a]	244	**16.8** [b]	301	16.6 [b]	0.009
Reference plausible	598	**16.6**	120	**15.9** [a]	211	**16.8** [b]	267	16.6 [b]	0.016
Adapted milk consumers	741	**15.6**	294	**15.0** [a]	262	**15.9** [b]	185	16.2 [b]	<0.001
Adapted milk consumers plausible	618	**15.6**	236	**14.9** [a]	224	**16.0** [b]	158	16.2 [b]	<0.001
**(%) Carbohydrates**									
Reference	707	**45.4**	162	**46,2**	244	45.2	301	45.1	0.176
Reference plausible	598	**45.3**	120	**46.1**	211	44.9	267	45.3	0.25
Adapted milk consumers	741	**46.7**	294	48.3 [a]	262	45.9 [b]	185	45.3 [b]	<0.001
Adapted milk consumers plausible	618	**46.7**	236	**48.6** [a]	224	45.6 [b]	158	45.3 [b]	<0.001
**(%) Fat**									
Reference	707	36.5	162	**36.2**	244	36.4	301	36.7	0.63
Reference plausible	598	36.5	120	**36.2**	211	36.6	267	36.5	0.84
Adapted milk consumers	741	35.9	294	**34.6** [a]	262	36.6 [b]	185	37.0 [b]	<0.001
Adapted milk consumers plausible	618	36.0	236	**34.5** [a]	224	36.8 [b]	158	37.1 [b]	<0.001

EI: Energy intake. Results are expressed in percentage of contribution to the total energy intake. *T*-test was used to evaluate differences by sex and age groups between the reference and adapted milk consumers and between plausible reporters, reference plausible and adapted milk consumers plausible (mean values, are shown in boldface type). ANOVA analysis was used to calculate differences among age groups (mean values; values with different superscript letters were significantly different). *p*-value <0.05 was considered statistically significant. *Data from a total sample of 1446 participants of the EsNuPI study have been used for the evaluation of misreporting, due to the lack of information on physical activity data of two study participants (*n* = 1 SRS; *n* = 1 AMS).

Supplementary Table 4. The adequacy to the European Food Safe Authority and Institute of Medicine recommendations for energy and protein intakes and percentages of samples that meet these criteria for fat and carbohydrates for the plausible sample by age group and sex for the Nutritional Study in Spanish Pediatric Population (EsNuPI) (n = 1448).

	**Reference plausible sample**									**Adapted milk consumers plausible sample**								
	**Total**		**1-<3 years**		**3-<6 years**		**6-<10 years**			**Total**		**1-<3 years**		**3-<6 years**		**6-<10 years**		
	**n**	**%**	**n**	**%**	**n**	**%**	**n**	**%**	***p***	**n**	**%**	**n**	**%**	**n**	**%**	**n**	**%**	***p***
**Adequacy to recommendations** [1]																		
**(%) Energy intake EFSA**	598	**111**	120	133 ^{a}	211	115 ^{b}	267	97.7 ^{c}	<0.001	618	**117**	236	134 ^{a}	224	114 ^{b}	158	97.0 ^{c}	<0.001
Boys	307	112	69	130 ^{a}	109	115 ^{b}	129	99.6 ^{c}	<0.001	314	113	119	128 ^{a}	111	110 ^{b}	84	95.5 ^{c}	<0.001
Girls	291	**110**	51	137 ^{a}	102	115 ^{b}	138	95.9 ^{c}	<0.001	304	**122**	117	140 ^{a}	113	118 ^{b}	74	98.8 ^{c}	<0.001
**(%) Energy intake IOM**	598	**80.6**	120	120 ^{a}	211	74.6 ^{b}	267	68.0 ^{c}	<0.001	618	**90.3**	236	118 ^{a}	224	75.2 ^{b}	158	69.1 ^{c}	<0.001
Boys	307	**79.6**	69	119 ^{a}	109	71.8 ^{b}	129	65.1 ^{c}	<0.001	314	**87.0**	119	118 ^{a}	111	70.8 ^{b}	84	64.4 ^{b}	<0.001
Girls	291	**81.8**	51	120 ^{a}	102	77.5 ^{b}	138	70.7 ^{c}	<0.001	304	**93.6**	117	120 ^{a}	113	79.4 ^{b}	74	74.3 ^{b}	<0.001
**(%) Proteins EFSA**	598	344	120	**376** ^{a}	211	409 ^{b}	267	279 ^{c}	<0.001	618	350	236	**351** ^{a}	224	397 ^{b}	158	283 ^{c}	<0.001
Boys	307	353	69	376 ^{a}	109	415 ^{b}	129	287 ^{c}	<0.001	314	342	119	336 ^{a}	111	394 ^{b}	84	283 ^{c}	<0.001
Girls	291	**335**	51	376 ^{a}	102	403 ^{a}	138	270 ^{b}	<0.001	304	**359**	117	365 ^{a}	113	400 ^{b}	74	284 $^{c a}$	<0.001
**(%) Proteins IOM**	598	348	120	**355** ^{a}	211	384 ^{b}	267	316 ^{c}	<0.001	618	337	236	**329** ^{a}	224	364 ^{b}	158	312 ^{a}	<0.001
Boys	307	**362**	69	365 ^{a}	109	394 ^{a}	129	334 ^{ab}	<0.001	314	332	119	320 ^{a}	111	358 ^{b}	84	317 ^{a}	0.001
Girls	291	333	51	342 ^{a}	102	373 ^{a}	138	299 ^{b}	<0.001	304	343	117	339 ^{a}	113	370 ^{ab}	74	306 ^{b}	<0.001
**Subjects meeting the recommendations** [2]																		
**(%) Carbohydrates EFSA** [2]	598	**50.7**	120	59.2	211	45.5	267	50.9	0.145	618	**57.6**	236	65.3 ^{a}	224	53.1 ^{b}	158	52.5 ^{b}	<0.001
Boys	307	**51.8**	69	60.9	109	45.9	129	51.9	0.070	314	**58.9**	119	68.1 ^{a}	111	55.9 ^{ab}	84	50.0 ^{b}	0.003
Girls	291	49.5	51	56.9	102	45.1	138	50.0	0.671	304	56.3	117	62.4	113	50.4	74	55.4	0.140
**(%) Carbohydrates IOM** [2]	598	**51.7**	120	60.0	211	46.9	267	51.7	0.142	618	**59.7**	236	69.5 ^{a}	224	54.5 ^{b}	158	52.5 ^{b}	0.002
Boys	307	52.4	69	60.9	109	47.7	129	51.9	0.331	314	61.1	119	73.1 ^{a}	111	56.8 ^{b}	84	50.0 ^{b}	0.007
Girls	291	50.9	51	58.8	102	46.1	138	51.4	0.325	304	58.2	117	65.8	113	52.2	74	55.4	0.096
**(%) Fat EFSA** [2]	598	**37.5**	120	27.5 ^{a}	211	36.5 ^{ab}	267	42.7 ^{b}	<0.001	618	**33.0**	236	28.0	224	36.2	158	36.1	<0.001
Boys	307	**38.1**	69	27.5	109	39.4	129	57.4	<0.001	314	**38.2**	119	32.8	111	41.4	84	58.3	<0.001
Girls	291	**36.8**	51	27.5	102	33.3	138	42.8	<0.001	304	**27.6**	117	32.1	113	31.0	74	29.7	<0.001
**(%) Fat IOM** [2]	598	**45.0**	120	50.0	211	46.4	267	41.6	<0.001	618	**47.4**	236	56.4 ^{a}	224	46.4 ^{ab}	158	35.4 ^{b}	<0.001
Boys	307	**47.6**	69	50.7	109	51.4	129	42.6	<0.001	314	**50.6**	119	56.3	111	51.4	84	58.3	<0.001
Girls	291	**42.3**	51	49.0	102	41.2	138	40.6	0.001	304	**44.1**	117	56.4 ^{a}	113	41.6 ^{ab}	74	28.4 ^{b}	<0.001

Results are expressed in percentage (%). Recommended daily intakes according to Europe Food Safety Authority (EFSA) and Institute of Medicine (IOM) [31,32]. [1] *t*-test was used to evaluate differences for the adequacy to the EFSA and IOM recommendations for energy and protein intakes by sex and age groups between the reference plausible and adapted milk consumers plausible sample (mean values, are shown in boldface type). ANOVA analysis was used to calculate differences for the adequacy to the EFSA and IOM recommendations for energy and protein intakes among age groups (mean values; values with different superscript letters were significantly different).[2] *Chi*-squared test was used to evaluate differences within the percentage of samples that meet these criteria for fat and carbohydrates recommendations by sex and age groups between the reference plausible and adapted milk consumers plausible sample (mean values, are shown in bold type). *Z*-test was used to calculate differences within the percentage of samples that meet these criteria for fat and carbohydrates recommendations among age groups (mean values; values with different superscript letters were significantly different).
